# Achievement of Sustained Net Plasma Heating in a Fusion Experiment with the Optometrist Algorithm

**DOI:** 10.1038/s41598-017-06645-7

**Published:** 2017-07-25

**Authors:** E. A. Baltz, E. Trask, M. Binderbauer, M. Dikovsky, H. Gota, R. Mendoza, J. C. Platt, P. F. Riley

**Affiliations:** 1 Google Inc., 1600 Amphitheatre Parkway, Mountain View, CA 94043 USA; 2 Tri Alpha Energy Inc., P.O. Box 7010, Rancho Santa Margarita, CA 92688 USA

## Abstract

Many fields of basic and applied science require efficiently exploring complex systems with high dimensionality. An example of such a challenge is optimising the performance of plasma fusion experiments. The highly-nonlinear and temporally-varying interaction between the plasma, its environment and external controls presents a considerable complexity in these experiments. A further difficulty arises from the fact that there is no single objective metric that fully captures both plasma quality and equipment constraints. To efficiently optimise the system, we develop the Optometrist Algorithm, a stochastic perturbation method combined with human choice. Analogous to getting an eyeglass prescription, the Optometrist Algorithm confronts a human operator with two alternative experimental settings and associated outcomes. A human operator then chooses which experiment produces subjectively better results. This innovative technique led to the discovery of an unexpected record confinement regime with positive net heating power in a field-reversed configuration plasma, characterised by a >50% reduction in the energy loss rate and concomitant increase in ion temperature and total plasma energy.

## Introduction

Complex systems with many parameters are common in biology, physics, engineering, geology, and social science. Understanding and optimising such systems is difficult due to time-dependent parameters combined with noisy and missing measurements. Research progress in these disciplines can be accelerated with new tools capable of efficient exploration.

Modern plasma fusion research is a good example of this kind of complex system. Typical fusion systems have many control and input parameters, such as voltages applied to magnets, electrodes, limiters, etc. To complicate matters, the equipment has uncontrolled drift: for example, varying conditions of the internal surfaces of the vacuum vessel may strongly affect an experimental run. Optimising plasma requires optimisation over many highly nonlinear and coupled parameters. Further, each experiment may take a long time. These factors preclude an effective mapping of the high-dimensional parametric space by typical one-variable-at-a-time methods^1^.

Two additional complications arise because plasma fusion apparatuses are experimental and one-of-a-kind. First, the goodness metric for plasma is not fully established and objective: some amount of human judgement is required to assess an experiment. Second, the boundaries of safe operation are not fully understood: it would be easy for a fully-automated optimisation algorithm to propose settings that would damage the apparatus and set back progress by weeks or months.

To increase the speed of learning and optimisation of plasma, we developed the Optometrist Algorithm. Just as in a visit to an optometrist, the algorithm offers a pair of choices to a human, and asks which one is preferable. Given the choice, the algorithm proceeds to offer another choice. While an optometrist asks a patient to choose between lens prescriptions based on clarity, our algorithm asks a human expert to choose between plasma settings based on experimental outcomes. The Optometrist Algorithm attempts to optimise a hidden utility model that the human experts may not be able to express explicitly.

The Optometrist Algorithm is motivated both by human choice experiments^2^ and by Monte Carlo (MC) optimisation methods^3^. Human choice experiments are typically used to elicit preference models in complex situations, such as in health care^4^. MC optimisation methods efficiently explore high-dimensional spaces to discover difficult-to-find optima.

Application of the Optometrist Algorithm to our plasma fusion problem yielded significant benefits. By deliberately exploring parameter space, we found plasma with better properties, such as stored energy and confinement times. The broader exploration of parameter space also improved the intuition of the human operators. Finally, the most significant outcome was the discovery of an unexpected plasma confinement regime, characterised by a factor of two reduction in the energy loss rate and resultant increase in the plasma temperature. The existence of this novel regime will significantly facilitate the ascent towards energy generation from fusion. It also validates the technique as a very useful tool in exploring complex, high-dimensional systems. The Optometrist Algorithm may be applicable to discovery of other scientific or engineering advances.

Our strategy of humans selecting between machine-generated settings is broadly applicable and differs from typical approaches to optimisation problems in the machine learning community, where strict figures of merit are automatically computed^5, 6^. As such, the Optometrist Algorithm provides for efficient optimisation in areas where computation of such metrics is not possible. The primary advantage of the Optometrist Algorithm is combining the best of machine and human: the human provides physics intuition while the machine searches high-dimensional space.

## Experimental Facility

**Figure 1:**
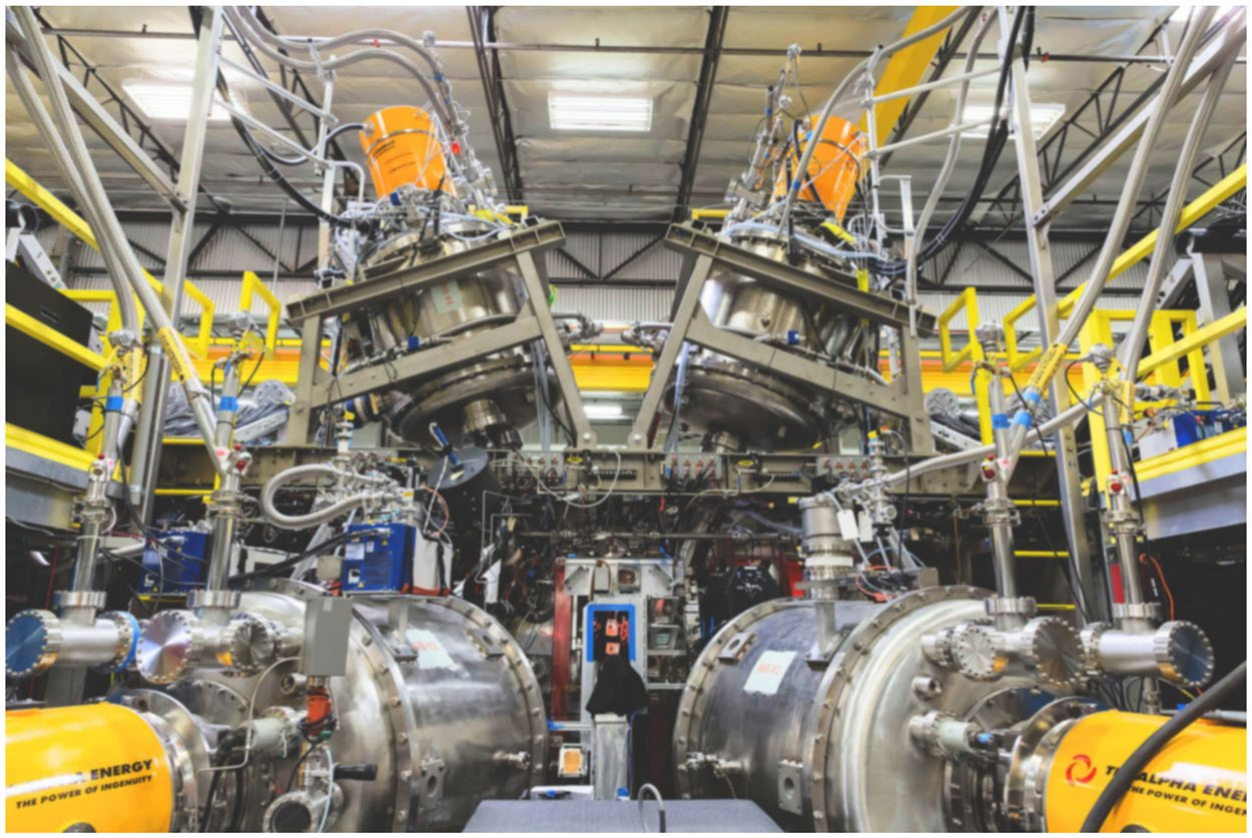
Central confinement chamber of C-2U, a plasma confinement experiment comprised of about 10,000 engineering control tags and over 1,000 physics diagnostics channels. Photo copyright belongs to Tri Alpha Energy Inc.

The experimental fusion apparatus at Tri Alpha Energy^7–10^, shown in Fig. 1, creates, confines, and heats a form of plasma called a Field-Reversed Configuration (FRC)^11, 12^. This experiment combines efficient use of magnetic fields (high *β*), large orbit ions (orbital radius comparable to system radius) for macro- and micro-stability, and a simply connected divertor (exhaust chamber) for safe removal of power with the goal of eventually enabling a compact power plant with reduced engineering complexity.

The C-2U machine^10^ on which this experiment was conducted is based on substantial upgrades to the C-2 facility^9^. The chief upgrade was to the neutral beam system. Particle energy was reduced from 20 keV to 15 keV, but total neutral current and power were increased in excess of 2.5 times that available in C-2. Total power over the 6 neutral beam injectors is more than 10 MW on C-2U. In addition, the injectors are angled inward at 20 degrees to better couple to plasma in the centre of the confinement region. Another upgrade was to the mirror plug magnets. They remained at 20 kG strength, but could maintain this field over the full 10 ms duration of the plasma shots, which was not possible in C-2. The final significant upgrade was to the end biasing systems for edge/stability control, now replaced with Gas Dynamic Trap (GDT) type plasma guns^13^, capable of biasing voltage and current at 1 kV and 3 kA, respectively. These upgrades together allowed the achievement of plasma sustainment, limited only by stored energy.

Though the engineering complexity of the C-2U device is greatly reduced when compared to other fusion research facilities, there are still thousands of system parameters that can be adjusted to maximise performance of the machine. These parameters include the control of a large number of subsystems, such as formation sections (where plasma is made) and high power particle beams (that heat plasma and reduce losses due to turbulence^14, 15^). Specific examples of important controlled parameters include voltages and timing of circuits that ionize and accelerate plasma, currents that set magnetic fields in different regions and voltages applied at the vessel ends to set boundary conditions.

## Exploratory Technique

The parameter space of C-2U has over one thousand dimensions. Quantities of interest are almost certainly not convex functions of this space. Furthermore, machine performance is strongly affected by uncontrolled time-dependent factors such as vacuum impurities and electrode wear. Under nominal operating conditions, plasma shots can be taken with a cadence of about eight minutes. Experiments are run over an eight-hour shift, producing up to 60 plasma shots per day. Given these facts, efficient optimisation of system performance appears to be a close to intractable problem. Nonetheless, we will show that the Optometrist Algorithm can overcome these difficulties. Pseudocode for the Optometrist Algorithm is given in Alg. 1, with a detailed explanation following. Mathematical details are given in the Methods section.

**Algorithm 1:**
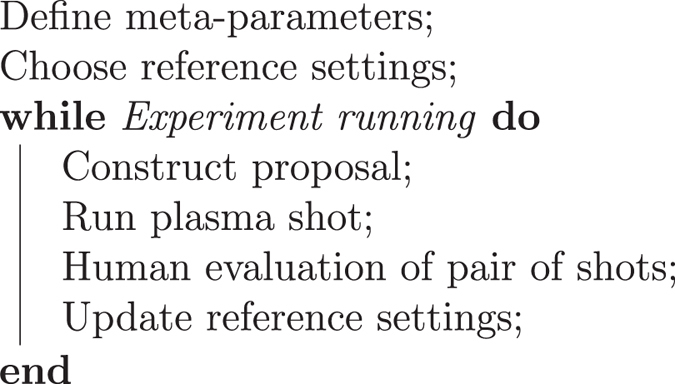
The Optometrist Algorithm.

While C-2U has thousands of configurable parameters, most subsystems can be effectively described by a much smaller space of so-called “meta-parameters” (MPs). We can adjust the effects of most subsystems with one or two meta-parameters, capturing the most important behaviours. In this work we used sets of less than 30 MPs, described in detail in the Methods section.

At the start of each day’s run, we repeat a small number (less than ten) of recent experiments. The human operator chooses the best among them as a reference. All following experiments are based on this initial reference.

New settings are chosen in the reduced space of MPs via a stochastic algorithm. Starting from the known operating point, we move in a random direction in MP-space, adjusting the parameters by a relative amount. As the values and units of the MP dimensions are heterogeneous, relative changes allow us to compare them in a meaningful way.

After a step in MP-space is taken, actual machine settings are derived by undoing the functional mapping for each MP. These settings are loaded into the control system and the shot is taken. Measurements of the experimental outcome are recorded and shown to a human expert in the next step.

The Optometrist Algorithm asks the human to compare two shots: the reference shot and the new shot just generated. The human expert can use his or her judgement about which shot is better, using all the available measurements that are shown in a visualisation panel. The human can choose one of the two shots as better, or rate them “about the same”. “About the same” is explained to the human as “50% likely to be as good, if the shot were to be repeated”. This allows for human judgement on partially successful experiments.

The human expert looks at visualisations related to plasma initial conditions, confinement times, and stability. The expert can choose based on multiple criteria, instead of just a pre-defined metric. Further, the goals of the optimisation can be changed during a run based on newly-discovered plasma behaviour.

The inclusion of expert oversight in parameter exploration is especially important given the large number of simultaneously-perturbed MPs. For instance, *a priori* identification of safe operating regimes cannot always be made for all combinations of machine parameters. As such, it is possible to set the machine to an unsafe state due to unanticipated nonlinear interactions between settings. This is not clear until the unsafe shot is actually run. Furthermore, safe settings may evolve over the course of experiments. For example, as part of the learning and experimentation process, deliberate hardware or procedural changes may be made to the machine or its operation. These changes can quickly impact vacuum and vessel wall conditions. For these reasons human oversight is crucial. The human expert can reject settings that yield both excellent plasma performance but also high likelihood of damaging the machine.

If the new shot is better than, or “about the same” as the old reference shot, it becomes the new reference shot. Otherwise, the settings are rejected and the reference shot remains the same. This strategy avoids getting stuck in local maxima. This is analogous to the Metropolis-Hastings acceptance algorithm for Monte Carlo optimisation^3, 16^, which accepts steps that degrade the optimisation function with some probability.

We note that a few of the best shots taken on a given day will be repeated on the next experiment day in the “Choose reference settings” phase. This strategy connects the exploration of MPs across experiment days, even permitting new exploration of previously dead ends.

## Results

We applied the Optometrist Algorithm to find useful and interesting parameter settings of the experimental plasma fusion apparatus shown in Fig. 1. For most of our experiment runs, we chose a set of approximately 20 meta-parameters. The full set of 29 MPs was only required if “North” and “South” subsystems needed independent adjustment, which was not common. We did not go below about 15 MPs because this was deemed the minimal set to describe the machine.

Over an experiment run of 3.5 weeks in September 2015, we used this technique over seven experiment days and 150 plasma shots. This does not include the shots used to establish reference settings at the start of each experiment day (totalling approximately 40 for this run).

**Figure 2:**
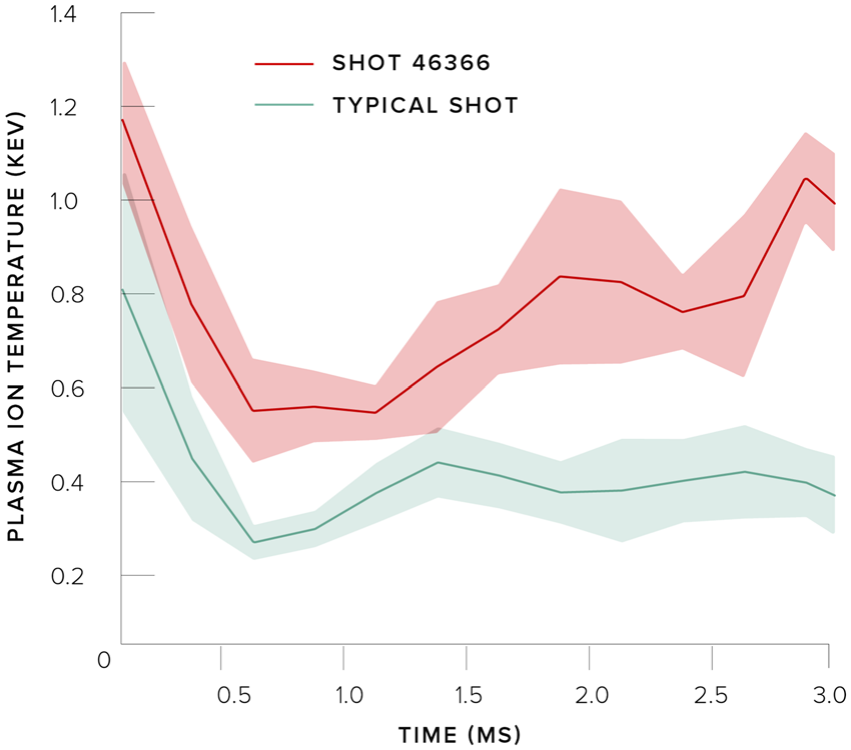
Plasma ion temperature climbs rapidly after 1 ms and reaches values of ∼1 keV by 3 ms into the discharge on Shot 46366. Typical temperatures are lower and show little to no rise in time.

**Figure 3:**
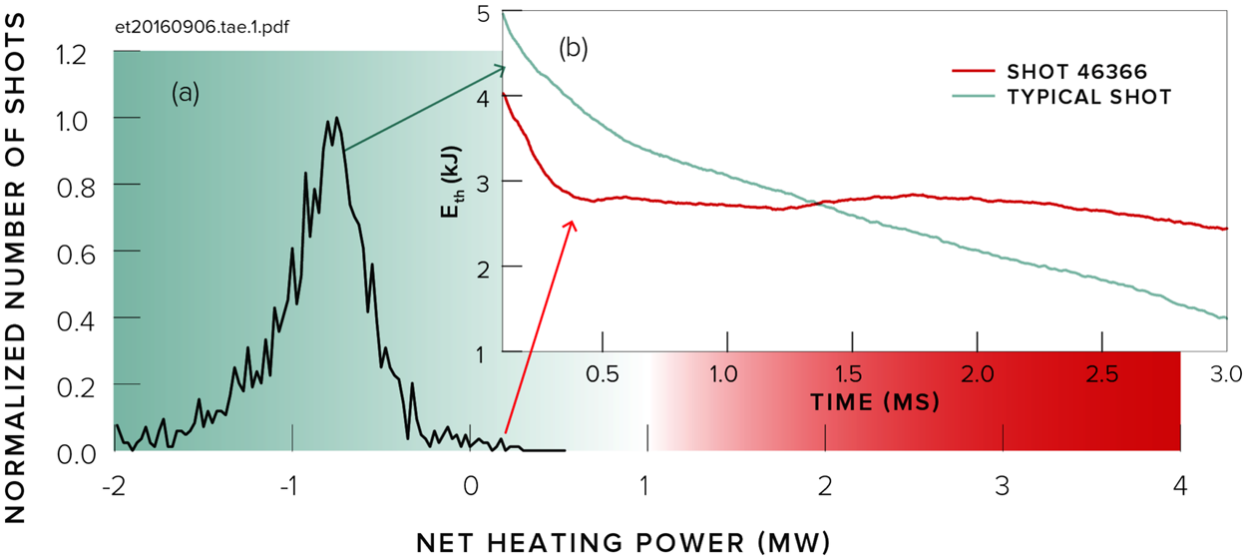
(**a**) Normalised distribution of the number of shots as a function of net heating power in C-2U based on over 1800 shots with at least 2 kJ of stored thermal energy (*E*_*th*_). Typical power losses are between 0.6 and 1 MW. Power is taken as the peak value in a rolling 1 ms time window between 1 and 3 ms. (**b**) Net power into the plasma exceeded 0.1 MW in Shot 46366 due to a reduction in cooling rates.

Before we started optimisation, the plasma would be stable for a small number of milliseconds, and the ion temperature would gradually decay with time. Typical plasma parameters for these conditions are shown in Table 1. We started using the Optometrist Algorithm to extend the stability of the plasma (ignoring the ion temperature). However, during the exploration of parameter space, the human operator noticed some experiments where the total plasma energy had brief increases. We then attempted to improve on this: we achieved a high and rapidly rising ion temperature (see Fig. 2) in addition to the total energy increase (see Fig. 3(b)) in Shot 46366. Both the absolute magnitude and the rate of rise of the temperature were significant, because one of the research goals at Tri Alpha Energy is to create hot ions.

**Table 1:** Average plasma parameters at 1.5 ms within the plasma radius from Shot 46366 as well as a ‘typical’ shot.

Parameter	Shot 46366	Typical Shot
*B*_*Ext*_, Confining B Field	750 G	760 G
*F*_*P*_, Poloidal Flux	5.6 mWb	4.8 mWb
*r*_ΔΦ_, Plasma Radius	33 cm	31 cm
$\bar{n}$, Average Density	1.9 * 10^13^ cm^−3^	2.7 * 10^13^ cm^−3^
*T*_*i*_, Ion Temperature	680 eV	370 eV
*T*_*e*_, Electron Temperature	125 eV	110 eV

This typical shot (47778) is the median of the distribution shown in Fig. 3 and has the most common time evolution of the plasma energy.

While the performance of this Shot 46366 was exemplary, on the same day a repeat and a single further Optometrist step each displayed rising total energy. Three days later, two direct repeats of 46366 again showed this behaviour. In addition, three days prior to taking Shot 46366, we took an Optometrist shot 46226 which was starting to display these excellent characteristics. The behaviour of this shot was repeated more than 10 times during the period of this particular experimental campaign, and before the objectives had to change. The best shots are summarised in Supplementary Table S1. Note in particular that the highest temperatures of the run were achieved by Shot 46366 and its repeat 46453.

Energy in the plasma is set by the difference between heating and loss rates, given by *dE*/*dt* = (*v*_*Heat*_ − *v*_*Loss*_)*E*. Throughout this experimental run, heating inputs (particle beams) were not changed compared with standard settings. Prior experimental analyses^9^ place the typical loss rate at ~1,250 s^−1^. Figure 3(a) shows the normalised distribution of the number of shots as a function of peak net heating power for the sequence of shots leading up to the discovery of the new confinement regime. As can be seen, the best shots achieved a period of net heating for the first time in the history of the apparatus. Comparing the difference in slopes, Δ*dE*/*dt*, between typical shot performance and the record setting series of shots shows that the loss rate, *v*_*Loss*_, was reduced by about 250 s^−1^ to 1000 s^−1^ or less.

A complete power balance analysis^17^ of these shots is beyond the scope of this paper, several conclusions can still be drawn based on prior knowledge of C-2/C-2U transport^18^. Ions and electrons primarily lose energy through convection, conduction and radiation. Typical total ion and electron cooling rates are about 700 s^−1^ and 3,500 s^−1^ (for *T*_*i*_ ≈ 4 * *T*_*e*_), respectively. Particle and radiated power loss rates remain unchanged when comparing values from the new record shots (*v*_*p*_ ≈ 400 s^−1^, *v*_*rad*_ ≈ 300 s^−1^) with typical values. Furthermore, ion heat conduction losses are insignificant (*v*_*q,i*_ ≈ 100 s^−1^) in C-2U. Therefore, the observed record confinement is due to a pronounced reduction in electron heat conduction (*v*_*q,e*_), from 2400 s^−1^ to 1200 s^−1^. This is a remarkable improvement in plasma confinement and a necessary step towards attaining higher plasma temperatures.

## Discussion

The problem of efficient exploration in many dimensions is certainly not unique to a plasma physics setting, and is applicable in many other disciplines. The highly nonlinear interactions innate to a magnetised fusion experiment, however, require systematic exploration. Changing one-variable-at-a-time is simply insufficient for the dimensionality of the problem. Blind randomisation is not necessarily the answer, because of concerns for damaging delicate pieces of equipment.

The Optometrist Algorithm complements model-based approaches by performing a thorough exploration of parameter space. We have built models of objective metrics on the data gathered from the Optometrist Algorithm. Another positive feature of the Optometrist Algorithm is that the optima of objective metrics may lie near the edge of the possible operating space. The tendency of humans to avoid such edges is counterbalanced by randomised exploration.

The most impactful benefit of the Optometrist Algorithm was the discovery of the unexpected regime of sustained net plasma heating. It is remarkable that this achievement was realised despite an *a priori* lack of knowledge of the causality or physics of the regime.

The Optometrist Algorithm is a solution to the common problem of understanding and optimising complex systems. Stochastic exploration combined with human-guided interpretation of results is a valuable tool that may solve a variety of difficult problems across modern science. We used the Optometrist Algorithm to advance understanding and performance of plasma fusion.

## Methods

### Metaparameters

Plasma experiments begin with a “formation” phase. First, gas is injected into the formation sections through puff valves. Typically there were 6 active puff valves, each with pressure and timing settings. The first two MPs unify these into a single injection pressure and time. The gas must then be ionized, controlled by three subsystems: the rotating magnetic field, the preionization, and the fast magnetic bias. Control of these systems is reduced to 3 voltages and 2 timing MPs. The ionized plasma is then linearly accelerated by a series of 17 very fast magnetic fields on each side of the device. The timings are adjustable individually (34 parameters, and another 34 for the activation of the crowbar system for safety), while the voltages are grouped into 16 banks. This complex system is reduced to a single unified voltage MP and two timing MPs. One of these is the “acceleration” MP where the timings are expanded or shrunk toward the zero point, for example the subset [0, 2, 4] → [0, 1.9, 3.8] μs.

Once the plasma is formed, it is maintained by the “equilibrium” systems. The dipole magnetic field is driven by 7 power supplies, which we chose to keep separate. At the ends of the confinement region, powerful mirror fields are specified by current and onset time. These can be specified separately for the two ends (4 MPs) or symmetrically (2 MPs). Finally, electrical biasing is typically achieved via plasma guns, which are controlled by gas pressure, timing, and two electrode voltages each. These can also be specified individually (8 MPs) or symmetrically (4 MPs). With these engineering choices, we reduce the dimensionality of the control space to <30, as summarised in Table 2. Most Optometrist runs adjusted only the symmetric set of 23, and we often chose to adjust a reduced set of 17 which left out the Mirror Field and Electrical Biasing parameters.

**Table 2:** Metaparameters for specifying experiments.

	Settings	Metaparameters
Formation: Voltages	34	4
Formation: Timings	80	4
Formation: Gas Pressure	6	1
Formation: Gas Timing	6	1
Equilibrium: Magnets	7	7
Equilibrium: Mirror Field	4	2 (4)
Equilibrium: Electrical Biasing	8	4 (8)
Totals	145	23 (29)

Values in parentheses indicate asymmetric operation, which was not commonly attempted or required. Note that settings for the equilibrium magnets were not believed to be further reducible. For many experiments there were additional reductions, e.g. not adjusting mirror fields and electrical biasing.

### Experiment proposals

We choose directions, $\vec{g}$, isotropically in the MP-space, drawing from an *N*-dimensional multivariate Student-t distribution^19^:

1 $$p(\vec{g})=\frac{{\rm{\Gamma }}((\nu +N)/\mathrm{2)}}{{\rm{\Gamma }}(\nu \mathrm{/2)(}\pi \nu {)}^{N\mathrm{/2}}}{[1+\frac{1}{\nu }{\vec{g}}^{T}\vec{g}]}^{-(\nu +N\mathrm{)/2}}$$

where *v* is the degrees of freedom on the t distribution. We choose *v* = *N*, as a compromise between a light-tailed Gaussian (*v* → ∞) and a very-heavy-tailed Cauchy (*v* = 1) distribution. A moderately heavy-tailed distribution of steps allows us to occasionally take very large steps (relative to the normal distribution) to better sample the unknown character of the performance of the machine. Taking heavy-tailed steps has been shown to be effective in Monte Carlo optimisation^20^. Notice, however, that the Optometrist Algorithm does not depend on the Student-t distribution.

The random direction at step *t*, ${\vec{g}}_{t}$, is multiplied by a step size, *η*_*t*_ and applied as a relative adjustment of the meta-parameters $\vec{x}$ to the reference shot ${\vec{x}}_{t-1}$:

2 $${\vec{x}}_{t}={\vec{x}}_{t-1}\,\circ \,\mathrm{(1}+{\eta }_{t}{\vec{g}}_{t})$$

where the step size *η*_*t*_ is adapted dynamically (as described, below).

### Step size adjustment

We adjust the step size dynamically to ensure that the probability of accepting the new reference shot is roughly controlled. In classical Monte Carlo, there is an optimal acceptance probability of 23.4%^21^. Accepting too many proposals means that exploration is not aggressive enough. Accepting too few proposals means not exploring enough or stepping too far. The step size *η*_*t*_ is adjusted via

3 $${\eta }_{t}\equiv A\exp ({\gamma }_{t})$$

4 $${\gamma }_{t}=\{\begin{array}{ll}{\gamma }_{t-1}+{\alpha }_{t}, & {\rm{if}}\,{\rm{human}}\,{\rm{prefers}}\,{\rm{new}}\,\text{shot};\\ {\gamma }_{t-1}-{\beta }_{t}, & {\rm{otherwise}}.\end{array}$$

The step size is also adjusted fractionally, starting from a baseline $A$. At each stage the step size is adjusted multiplicatively by exp(*γ*_*t*_) which starts at 1. The choice of *α*_*t*_ and *β*_*t*_ set the probability of an accepted step to be:

5 $${P}_{{\rm{accepted}}}=\frac{{\beta }_{t}}{{\alpha }_{t}+{\beta }_{t}}$$

We choose *β*_*t*_ = 0.5*α*_*t*_ to keep an acceptance probability of approximately 33%.

### Practical implementation

As discussed previously, the average shot cadence is eight minutes. Typically several minutes elapse before the first-line data analysis is complete. Only then can the expert human operator make an evaluation of the shot’s performance. The delay of the data analysis leaves only two or three minutes to generate and input a new proposal into the control system. To remove this bottleneck, we construct a decision tree of proposals, starting from known reference settings. This can be loaded into the control system in advance. The human operator can then simply traverse the decision tree of shot settings, at each step selecting the branch according to the choice made about the previous shot. In practice, we load binary trees 9 levels deep consisting of 512 shots with all settings predetermined, thereby staying over an hour ahead of actual experiments. We also explored higher order trees, requesting that the expert human operator provide more than a single binary decision, but this method did not provide additional benefits.

## Electronic supplementary material

Supplementary Dataset 1
